# Improving consumption rate estimates by incorporating wild activity into a bioenergetics model

**DOI:** 10.1002/ece3.2027

**Published:** 2016-03-04

**Authors:** Stephanie Brodie, Matthew D. Taylor, James A. Smith, Iain M. Suthers, Charles A. Gray, Nicholas L. Payne

**Affiliations:** ^1^ School of Biological, Earth and Environmental SciencesEvolution and Ecology Research CentreUniversity of New South WalesSydneyNSW2052Australia; ^2^ Sydney Institute of Marine ScienceMosmanNSW2028Australia; ^3^ New South Wales Department of Primary IndustriesPort Stephens Fisheries InstituteLocked Bag 1Nelson BayNSW2315Australia; ^4^ WildFish ResearchGrays PointNSW2232Australia; ^5^ National Institute of Polar ResearchTachikawaTokyo190‐8518Japan

**Keywords:** Acceleration, daily energy expenditure, dynamic body activity, ecological energetics, energy budget, field metabolic rate, predatory impact

## Abstract

Consumption is the basis of metabolic and trophic ecology and is used to assess an animal's trophic impact. The contribution of activity to an animal's energy budget is an important parameter when estimating consumption, yet activity is usually measured in captive animals. Developments in telemetry have allowed the energetic costs of activity to be measured for wild animals; however, wild activity is seldom incorporated into estimates of consumption rates. We calculated the consumption rate of a free‐ranging marine predator (yellowtail kingfish, *Seriola lalandi*) by integrating the energetic cost of free‐ranging activity into a bioenergetics model. Accelerometry transmitters were used in conjunction with laboratory respirometry trials to estimate kingfish active metabolic rate in the wild. These field‐derived consumption rate estimates were compared with those estimated by two traditional bioenergetics methods. The first method derived routine swimming speed from fish morphology as an index of activity (a “morphometric” method), and the second considered activity as a fixed proportion of standard metabolic rate (a “physiological” method). The mean consumption rate for free‐ranging kingfish measured by accelerometry was 152 J·g^−1^·day^−1^, which lay between the estimates from the morphometric method (*μ *= 134 J·g^−1^·day^−1^) and the physiological method (*μ *= 181 J·g^−1^·day^−1^). Incorporating field‐derived activity values resulted in the smallest variance in log‐normally distributed consumption rates (*σ *= 0.31), compared with the morphometric (*σ *= 0.57) and physiological (*σ *= 0.78) methods. Incorporating field‐derived activity into bioenergetics models probably provided more realistic estimates of consumption rate compared with the traditional methods, which may further our understanding of trophic interactions that underpin ecosystem‐based fisheries management. The general methods used to estimate active metabolic rates of free‐ranging fish could be extended to examine ecological energetics and trophic interactions across aquatic and terrestrial ecosystems.

## Introduction

Assessing an organism's ecological requirements and constraints is central to predicting species distributions (Elith and Leathwick 2009), community dynamics (Byrom et al. 2014), and trophic interactions (Young et al. 2015). Consumption is the most fundamental trophic interaction, and it can be calculated from diet analyses (Selch and Chipps 2007; Hughes et al. 2014), body composition (Coltrane et al. 2011; Pavlova et al. 2014), empirical regressions (Pauly 1989), and bioenergetics models (Kitchell et al. 1977; Hansen et al. 1993). Bioenergetics models estimate consumption rates based on the energetic requirements for growth, metabolism, reproduction, and waste (Olson and Boggs 1986). Metabolism, in turn, can be partitioned into the energy required for basal processes (resting/maintenance metabolic rate) and the energy required for activity (active metabolic rate). The energy required for activity can represent a large and variable proportion of an organism's energy budget (Boisclair and Sirois 1993; Briggs and Post 1997; Essington 2003; Halsey et al. 2015).

A central challenge in bioenergetics modeling is measuring the contribution of activity to an animal's energy budget. Hansen et al. (1993) discussed the future application of bioenergetics models and highlighted the need to account for the metabolic costs of activity. Despite the recent advances in technology that can help predict metabolic rate, few studies have linked metabolism and activity rates of free‐ranging animals with estimates of consumption or predatory impact (e.g., Halsey et al. 2008a; Payne et al. 2011). Consumption is the essential element of trophic ecology, from single‐species models to ecosystem models (Kinzey and Punt 2009; Taylor et al. 2013a). Such models will be improved when the activity component of individual consumption is derived from field data, under local conditions and for specific species, and incorporates the real variation in wild activity.

Activity of free‐ranging organisms can be measured in a variety of ways, using methods such as heart rate biotelemetry (Butler et al. 2004; Halsey et al. 2008b), doubly‐labeled water (Speakman 1998; Shaffer 2011), electromyography (EMG; Briggs and Post 1997; Cooke et al. 2004b), or body acceleration (Cavagna et al. 1963; Cooke et al. 2004a; Halsey et al. 2009). However, measuring activity on aquatic animals precludes many of the typical methods. For instance, applications of doubly‐labeled water and heart rate methods to aquatic species result in high inaccuracies due to high water flux through fish (Nagy and Costa 1980), and highly variable cardiac stroke volume with physiological state and environmental factors (Thorarensen et al. 1996). EMG has been shown to be effective for examining fish body activity, but the surgical implantation of electrodes requires high precision and accuracy (Cooke et al. 2004b). Body acceleration directly relates to energy expenditure (Cavagna et al. 1963; Halsey et al. 2008b) and can indicate the allocation of energy to different animal behaviors (Payne et al. 2013). Advances in acoustic telemetry have allowed remote measurement of body activity through accelerometers linked with remote tracking, thus allowing field‐based assessment of aquatic animal behavior (Murchie et al. 2011; Wilson et al. 2013). Quantifying the metabolic costs of body activity is important in understanding animal ecology, yet the energetic costs of field‐derived body activity have not been integrated into estimates of consumption rates.

This study describes how measured activity rates in free‐ranging animals can be incorporated into bioenergetics models for estimating consumption rate. The method used laboratory calibrations and field activity data to estimate field metabolic rate, and then incorporated this into a bioenergetics model to estimate consumption rate. This method is presented using a marine pelagic predator, yellowtail kingfish *Seriola lalandi* (Valenciennes 1833), as a study species. Little is known about the trophic ecology of kingfish, and consumption rate estimates will be useful for determining the trophic level of kingfish and associated trophic interactions. The field‐derived activity method was compared with two traditional bioenergetics methods to evaluate the contribution of field‐derived activity to consumption rate estimates.

## Materials and Methods

### Estimating field metabolic rate via accelerometry

Estimating field metabolic rates required two steps, the first involved laboratory experiments to determine active metabolic rate, and the second step required sampling activity data from wild kingfish. Laboratory experiments were conducted to describe the relationship between activity (dynamic body acceleration), swimming speed, and metabolic rate. These experiments were necessary to measure active metabolic rate, and involved measuring the oxygen consumption rate of wild caught kingfish implanted with activity tags. Seven juvenile kingfish (465–660 mm TL; mean 576.4 mm TL) were used in the calibration and all were captured at South Solitary Island, Australia (30°12′7.20″S; 153°15′57.60″E). Fish were held in a flow‐through aquarium system at the National Marine Science Centre, Southern Cross University, Australia, maintained at 24 ± 1°C, and fed on a diet of fresh and frozen cephalopod and teleost food. Fish were internally tagged with an acoustic transmitter that measures activity (Vemco, Halifax, Nova Scotia, Canada, Model V13AP‐H) using standard surgical techniques (Taylor et al. 2013b). Activity (m·sec^−2^) was determined by calculating the root mean square of triaxial acceleration (5 Hz) measured over a 20‐sec period. This calculation occurred onboard the tag with a single activity value acoustically transmitted within a random period of 50–100 sec (there was no archival logging of data).

Fish were allowed to recover from surgery for a minimum of 5 days and were fasted for 12 h before being introduced to a Brett‐type swimming respirometer (Brett 1964) at 24 ± 1°C (690 L, swim chamber dimensions 40 cm diameter × 120 cm length). Five days was considered a sufficient period for full recovery based on the observation of fish behavior. The experimental temperature was the mean ocean temperature at South Solitary Island during late summer – the same period as the calibration trials (NSW Marine Park Authority). Prior to each swimming trial, fish were acclimated to the respirometer for 3 h at the slowest water velocity that maintained steady swimming. Each fish was swum at between five and eight incremental speeds (0.1–0.8 m·sec^−1^) for 15 min at each speed. The respirometer was completely sealed from any sources of atmospheric air and dissolved oxygen concentrations were measured using a Hach oxygen meter (Hach HQ40d Loveland, Colorado, USA). Oxygen consumption rate during each swimming trial was measured as a metric of metabolic rate. After each swimming trial, fresh seawater was flushed through the respirometer to return oxygen levels to ambient concentrations. If dissolved oxygen levels in the respirometer fell below 80% saturation during a swimming trial, the trial was paused and the respirometer flushed with fresh seawater. An acoustic hydrophone (Vemco, Model VR100) was used to record the acoustic transmissions of activity data during each swimming trial. Fish were returned to the aquaria once swimming trials were completed.

Animals represented >10% of the cross‐sectional area of the swim chamber, so blocking correction factors were applied to correct swimming speed (Bell and Terhune 1970). Oxygen consumption rates were converted to mass‐specific metabolic rates using an exponent of 0.79; the mean scaling exponent for both resting metabolism in teleosts (Clarke and Johnston 1999) and swimming metabolism in sharks (Payne et al. 2015). Linear mixed‐effects modeling was performed to analyze laboratory trials using the “lme4*”* package (Bates et al. 2015) in R statistical computing (v3.0.3; R Core Development Team 2014). Mass‐specific oxygen consumption (*MR*_*A*_) was expressed as a function of activity according to (eq. (1)):

(1) $${MR}_{A}=s\;e{\;}^{b\;\;Act},$$

where *s* is the intercept, and *b* the coefficient of the relationship between metabolic rate and activity (*Act*). An exponential form for equation (1) was selected based on a comparison of corrected Akaike's Information Criteria (AICc) values between exponential and linear forms (Table 1). Equation (1) was solved by log_e_‐transforming *MR*_*A*_ and using a linear mixed‐effects model of the form (eq. (2)):

(2) $$\log\left({MR}_{A}\right)=\log\left(s\right)+b\;Act+{ID}_{rand},$$

where individual (*ID*_*rand*_) was included as a random factor to account for correlation in measurements within individuals. Analysis of the laboratory trials also expressed mass‐specific oxygen consumption (*MR*_*S*_) as a function of swimming speed according to (eq. (3)):

(3) $${MR}_{S}=r\;e{\;}^{z\;\;SS},$$

where *r* is the intercept, and *z* the exponent of the exponential relationship between metabolic rate and swimming speed (SS). Equation (3) was solved by log_e_‐transforming *MR_S_* and using a linear mixed‐effects model of the form (eq. (4)):

(4) $$\log\left({MR}_{S}\right)=\log\left(r\right)+z\;SS+{ID}_{rand}.$$

**Table 1:** Comparison of exponential and linear forms of the relationship between metabolic rate and activity (eq. (1)). Corrected Akaike's Information Criteria and model weights are given for each model

Form	Equation	AICc	ΔAICc	Weight
Exponential (eq. (1))	*MR*_*A*_ = *s e* ^*b Act*^	41.44	0	1
Linear	*MR*_*A*_ = *b Act* + *s*	464.37	422.93	1E‐92

The second step in estimating field metabolic rates required sampling activity from wild kingfish. Activity was measured in situ on the same experimental kingfish (*n* = 7) after they were released at their site of capture, South Solitary Island. South Solitary Island had three established acoustic receivers, which were used to record the movements and activity of these seven kingfish from March to May 2013. Prior to release, these fish were given at least 14 days to recover from surgery to reduce any effects of tagging on fish behaviour. After release, the first 24 h of field data was not included in the analysis to reduce any potential behavioral bias associated with reacclimating to the natural environment. Hourly bottom temperature (mean 21 m depth) was recorded by temperature loggers attached to each acoustic receiver mooring. These temperatures were used to estimate the environmental temperature of kingfish during the monitoring period. Any acoustic transmissions recorded in temperatures outside the temperature range of the laboratory calibrations (24 ± 1°C) were excluded to avoid any uncertainty associated with the temperature dependence of the activity–metabolism relationship (Halsey et al. 2015).

### Estimating consumption rate

The consumption rate of individual kingfish, incorporating the field‐derived and laboratory‐calibrated estimates of activity, was calculated using a bioenergetics approach based on the energy balance model of Olson and Boggs (1986) (eq. (5)):

(5) $$C=\frac{AMR+G}{A},$$

where *C* is consumption rate (J·g^−1^·day^−1^), *AMR* is the active metabolic rate (J·g^−1^·day^−1^), and *G* the energy allocated to fish growth (J·g^−1^·day^−1^; Table 2). *A* represents the proportion of total energy consumed lost due to assimilation, egestion, and excretion (Rice et al. 1983). Active metabolic rate (*AMR*) was calculated by converting field activity values to metabolic rate using the relationship derived from laboratory calibrations (eq. (1)). Field activity data from the seven kingfish were not uniformly distributed across a 24‐h period due to diel behavior of kingfish. To account for this, activity was split into day periods (*Act_day_*; 0700–1800 h) and night periods (*Act_night_*; 1900–0600 h). The mean hourly activity value within each period was converted to metabolic rate, based on equation (1) derived from the laboratory calibrations, and multiplied by the hours in that time period (eq. (6)):

(6) $$AMR=\left(\left(se^{bAct_{day}}\right)h_{d}+\left(se^{bAct_{night}}\right)h_{n}\right)Oxy,$$

where *s* is the intercept, *b* the exponent of the derived relationship between metabolic rate and activity (eq. (1); Table 2), *h*_d_ is the number of hours in the day period (0700–1800 h), *h*_*n*_ is the number of hours in the night period (1900–0600 h), and *Oxy* is the oxy caloric coefficient of 14.14 J·mgO_2_^−1^ (Elliott and Davison 1975). The energetic costs associated with reproduction were assumed to be negligible as the individuals monitored were juveniles.

**Table 2:** Summary of derived or literature mean parameter values and standard deviations (SD) used in calculating consumption rates of kingfish

Parameter symbol	Parameter descrption	Value	SD	Units	Equation	Source
*A*	Assimilation, egestion, and excretion costs	0.685	0.0175	–	(5)	Rice et al. (1983)
*Act_day_*	Mean hourly transmitted activity value during the day	1.582	0.1397	m·sec^−2^	(5)	Derived
*Act_night_*	Mean hourly transmitted activity value during the night	0.808	0.0647	m·sec^−2^	(5)	Derived
*Ac*	Coefficient of swim speed regression	0.3478	0.0782	–	(8)	Sambilay (1990)
*AM*	Activity multiplier	2	–	–	(5)	Winberg (1956) and Kitchell et al. (1977)
*AR*	Aspect Ratio	1.756	0.26	–	(8)	Measured
*b*	Slope of relationship between metabolic rate and activity	1.0907	0.1901	–	(1), (2), (6)	Derived, Figure 1B
*Fj*	Average energy density of *Katsuwonis pelamis*,* Thunnus albacares*, and *Pomatomus saltatrix*	6210	220	J·g^−1^	(7)	Boggs and Kitchell (1991) and Hartman and Brandt (1995)
*Gv*	von Bertalanffy growth rate, converted to mass.	2.158	0.2158^a^	g^−1^·day^−1^	(7)	Derived
*h*_d_	Number of hours in the day period (0700–1800 h)	12	–	–	(6)	Measured
*h*_*n*_	Number of hours in the night period (1900–0600 h)	12	–	–	(6)	Measured
*Int*	Intercept of swim speed regression	−0.828	0.2299	–	(8)	Sambilay (1990)
*Lc*	Coefficient of swim speed regression	0.6196	0.0562	–	(8)	Sambilay (1990)
*Oxy*	Oxy calorific coefficient	14.14	0.135	J·mgO_2_^−1^	(5)	Elliott and Davison (1975)
*Q*_10_	Rate at which standard metabolism increases with a 10°C increase	1.5536	0.15536^a^	–	(10)	Pirozzi and Booth (2009)
log(*r*)	Intercept of relationship between metabolic rate and swimming speed	4.6423	0.1867	–	(3), (4)	Derived, Figure 1A
*R*_a_	Intercept of relationship between metabolic rate and mass	0.0067	0.00067^a^	–	(9)	Clarke and Johnston (1999)
*R*_b_	Slope of relationship between metabolic rate and mass	−0.21	0.11	–	(9)	Clarke and Johnston (1999)
log(*s*)	Intercept of relationship between metabolic rate and activity	4.2387	0.1777	–	(1), (2), (6)	Derived, Figure 1B
*SL*	Standard length	49	5.745	cm	(8)	Measured
*Speed*	Routine swimming speed	0.7245	0.2376	m·sec^−1^		Derived
*T*	Temperature	23.8	0.53	°C	(10)	Measured
*W*	Mass of fish	1816.3	621.3	g	(7)	Measured
*z*	Slope of relationship between metabolic rate and swimming speed	1.3098	0.3843	–	(3), (4)	Derived, Figure 1A

a Indicate parameters that have an assumed SD 10% of the mean.

The energy allocated to daily somatic growth (*G*; J·g^−1^·day^−1^) was determined by (eq. (7)):

(7) G=(Gv/W)×Fj,

where *Gv* (g·day^−1^; Table 2) is the daily growth rate, *W* is mean weight (g), and *Fj* is the energy density of somatic tissue (J·g^−1^; Table 2). *Gv* for kingfish is the von Bertalanffy growth rate (*t*_0_ = −4.4, *k *=* *0.54, *L*_*∞*_ = 184) (Stewart et al. 2004) converted to mass from a kingfish length–weight regression provided by Stewart et al. (2001). Growth rates decrease with increasing fish age, so the mean fish length of experimental kingfish (51 cm FL; 1.62 years) was used in calculating daily growth rate. There are no estimates of energy density for yellowtail kingfish, so the average energetic content of physiologically similar species (*Katsuwonus pelamis*,* Thunnus albacares*,* Pomatomus saltatrix*) was used due to similarities in ecology and trophic levels (Boggs and Kitchell 1991; Hartman and Brandt 1995).

Consumption rates of kingfish were also calculated using two traditional methods. These methods estimate consumption as above (eq. (5)), but differ in how they calculate active metabolic rate. The calculations for active metabolic rate are presented for each method, and these values are entered into equation (5) as *AMR* to estimate kingfish consumption. The first method, hereafter referred to as the morphometric model, requires relationships between swimming speed and fish length, and swimming speed and metabolic rate (eq. (3)), to estimate active metabolic rate. Morphological traits have been used for obligate ram ventilators such as tuna by using the minimum swimming speed required for hydrodynamic lift as a metric for routine swimming speed (Magnuson 1973; Essington 2003). For the morphometric method, routine swimming speed was estimated using an empirical multiple regression relating kingfish swimming speed to their standard length and aspect ratio of their caudal fin (Sambilay 1990) (eq. (8)):

(8) $$\log_{10}Speed=Int+Lc\log_{10}SL+Ac\log_{10}AR,$$

where *Speed* is routine swimming speed (km·h^−1^), *Int* is the intercept of the regression, *Lc* is a coefficient, *SL* is standard length (cm), *Ac* is a coefficient, and *AR* is aspect ratio of the caudal fin (Table 2). *AR* is the ratio of the height and surface area of the caudal fin, and for kingfish was calculated from photographs (*n* = 5; 350–670 mm TL) using ImageJ software (Abràmoff et al. 2004). The mean standard length (*SL*) of the seven experimental kingfish was used in equation (8). The derived routine swimming speed was used to determine active metabolic rate (mgO_2_ kg^−1^·h^−1^) using the relationship derived from laboratory calibrations (*MR_S_*; eq. (3)). This value of active metabolic rate was converted into J·g^−1^·day^−1^ and entered as *AMR* in equation (5).

The second method, hereafter referred to as the physiological model, used species‐specific physiological estimates of metabolic rate within the bioenergetics formula of the software Fish Bioenergetics 3.0 (Hanson et al. 1997). This a common approach for estimating consumption rates and relies upon sampling physiological parameters of the species of interest. The function for active metabolic rate (*R;* gO_2_ g^−1^·day^−1^) is (eq. (9)):

(9) $$R=R_{a}W^{R_{b}}Temp\;\;AM,$$

where *R*_a_ and *R*_b_ are the intercept and slope of the allometric function for mass‐specific standard metabolic rate, *W* (g) is the wet weight of the predator, *Temp* is a temperature dependence function (see below), and *AM* is an activity multiplier that scales standard metabolic rate to active metabolic rate (Table 2). *R*_a_ and *R*_b_ are coefficients from a general teleost fish allometry function estimated from Clarke and Johnston (1999). The mean weight (*W*) of the seven experimental kingfish was used in equation (9). The activity multiplier (*AM*) was set at 2 to estimate the active metabolic rate of wild fish (Winberg 1956). The value of the activity multiplier is subjective but consistent with other studies (Meskendahl et al. 2010; Whiterod et al. 2013; Johnson et al. 2015), and is based on the assumption that active metabolic rate is a fixed proportion of standard metabolic rate. This fixed proportion is likely to introduce unknown error into consumption rate estimates, but is included here due to its prevalence in the literature. The temperature dependence function *Temp* is calculated as (eq. (10)):

(10) $$Temp=e^{(\left(\ln\left(Q_{10}\right)/10\right)T},$$

where *Q*_10_ is the increase in standard metabolism with an increase in 10°C, and *T* is temperature (°C). In this study, *T* was the mean temperature from the sampling period at which acoustic transmissions were recorded. Within this sampling period, only temperatures within the boundary of the laboratory calibrations (24 ± 1°C; Table 2) were included in calculating the mean. This was done to ensure that the mean and variance of *T* matched the observations included in the accelerometer method. The value for *R* (eq. (9)) was converted to units of J·g^−1^·day^−1^ and entered as *AMR* in equation (5).

### Sensitivity analysis and consumption rate simulations

A sensitivity analysis was undertaken to determine the relative effect of each parameter on consumption rate estimates and included all parameters in each of the three models (accelerometry, morphometric, and physiological models). In this analysis, Monte Carlo simulations were used to select unique parameter values for each model run, where parameter values were randomly varied to be either the mean value or the mean value ±10%. Simulations were continued until the variance in consumption rate estimates stabilized (10,000 iterations). The random parameter values and model outputs were then standardized following Kleijnen (1997) to allow for a comparison of parameter relative importance. A multiple linear regression of standardized simulation results was used to express consumption as a function of parameter values, and the resulting parameter coefficients represent the relative importance of parameters in determining consumption rates (Smith et al. 2012).

The variance, or uncertainty, of parameter values was incorporated into final consumption rate estimates for each of the three models using Monte Carlo simulations in MATLAB (Mathworks Natick, Massachusetts, USA; Taylor et al. 2013a). Consumption rate simulations accounted for the uncertainty in parameter estimates by sampling parameter values from normal probability distributions. Probability distributions were developed using mean and variance estimates from laboratory calibrations as well as published sources (Table 2). Parameters that did not have published sources of error were allocated a standard deviation equal to 10% of the mean. Each model simulation sampled a value from each parameter's normal probability distribution, with simulations continued until the variance in consumption rate estimates stabilized (10,000 iterations). This produced a probability distribution of consumption rate estimates for each model, with a log‐normal curve fitted to simulation outputs.

## Results

### Field metabolic rate via accelerometry

**Figure 1:**
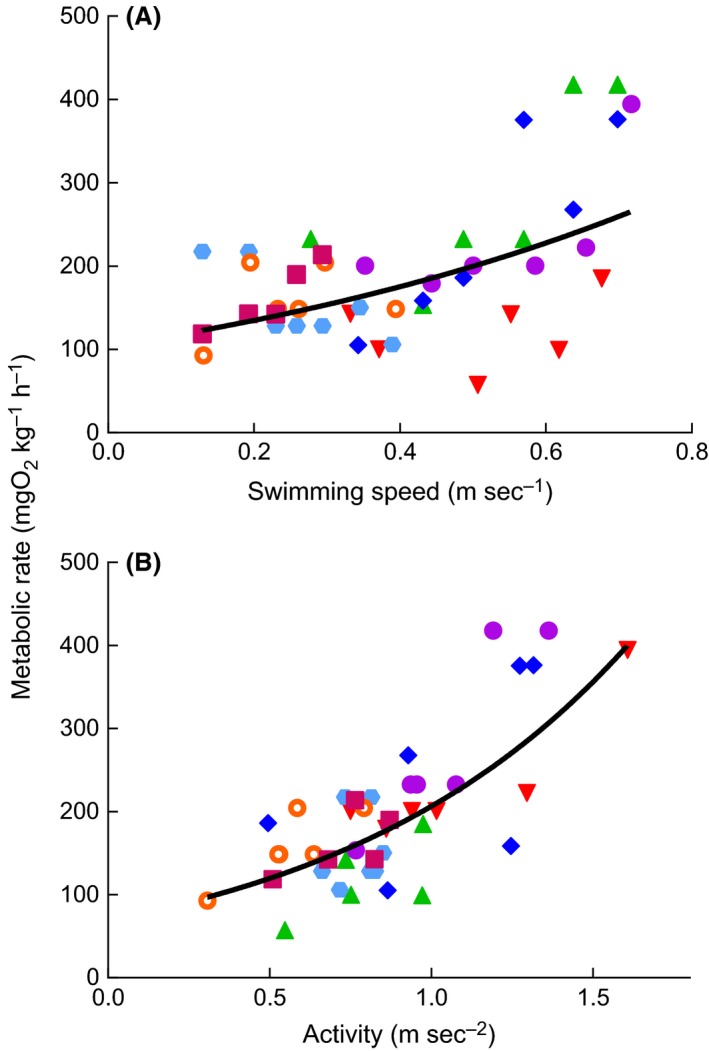
Relationship between metabolic rate and swimming speed (A) and activity (B) for kingfish (*n* = 7, symbols and colors represent individual fish). Solid lines represent the exponential relationship derived from the linear mixed‐effects models (eqs. (1) and (3)).

**Figure 2:**
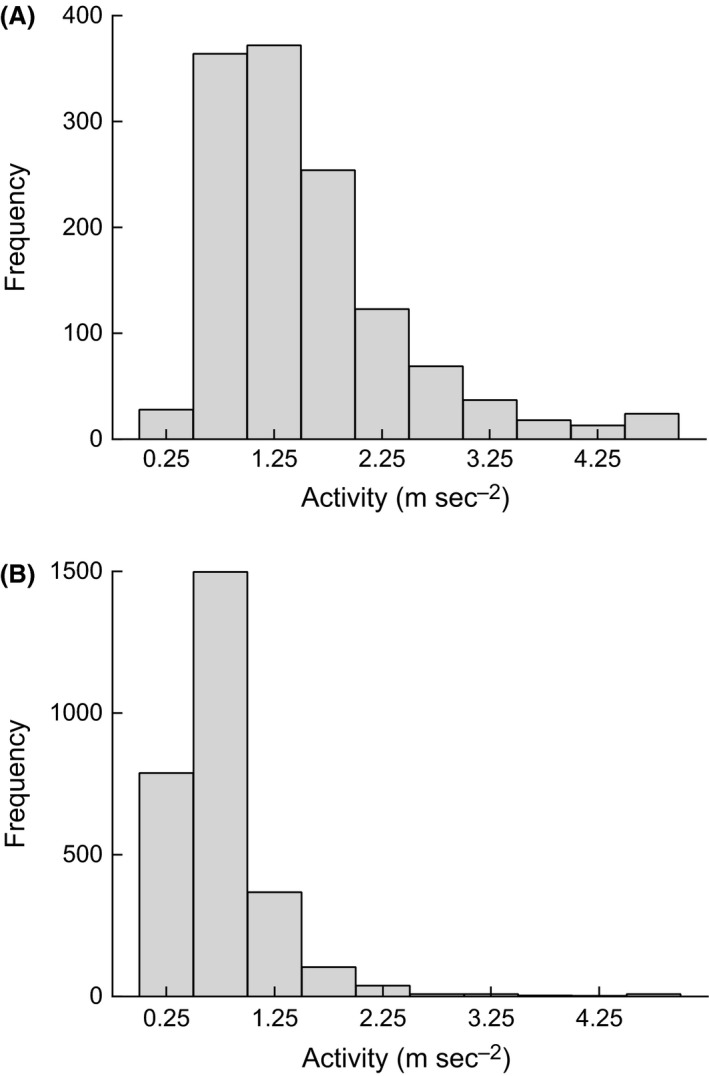
Frequency distribution of field activity values during the day (A) and night (B) for wild kingfish (*n* = 7) monitored in situ for 72 days at temperatures 24 ± 1°C.

Throughout the duration of the laboratory swimming trials, oxygen consumption by the experimental kingfish declined linearly. Exponential models best described the increase in kingfish metabolic rate (mgO_2_ kg^−1^·h^−1^) with swimming speed (Fig. 1A), and activity (Fig. 1B). All seven experimental kingfish were detected within the South Solitary Island acoustic array for durations between 4 and 69 days after they were released (mean 44 days’ detection duration). Acceleration values were transmitted frequently within this period, with a mean 60 detections per day. Kingfish showed strong diel behavior, with higher activity values during the day (1.58 ± 0.14 m·sec^−2^; Fig. 2A) than at night (0.81 ± 0.06 m·sec^−2^; Fig. 2B).

### Consumption rate parameters

**Figure 3:**
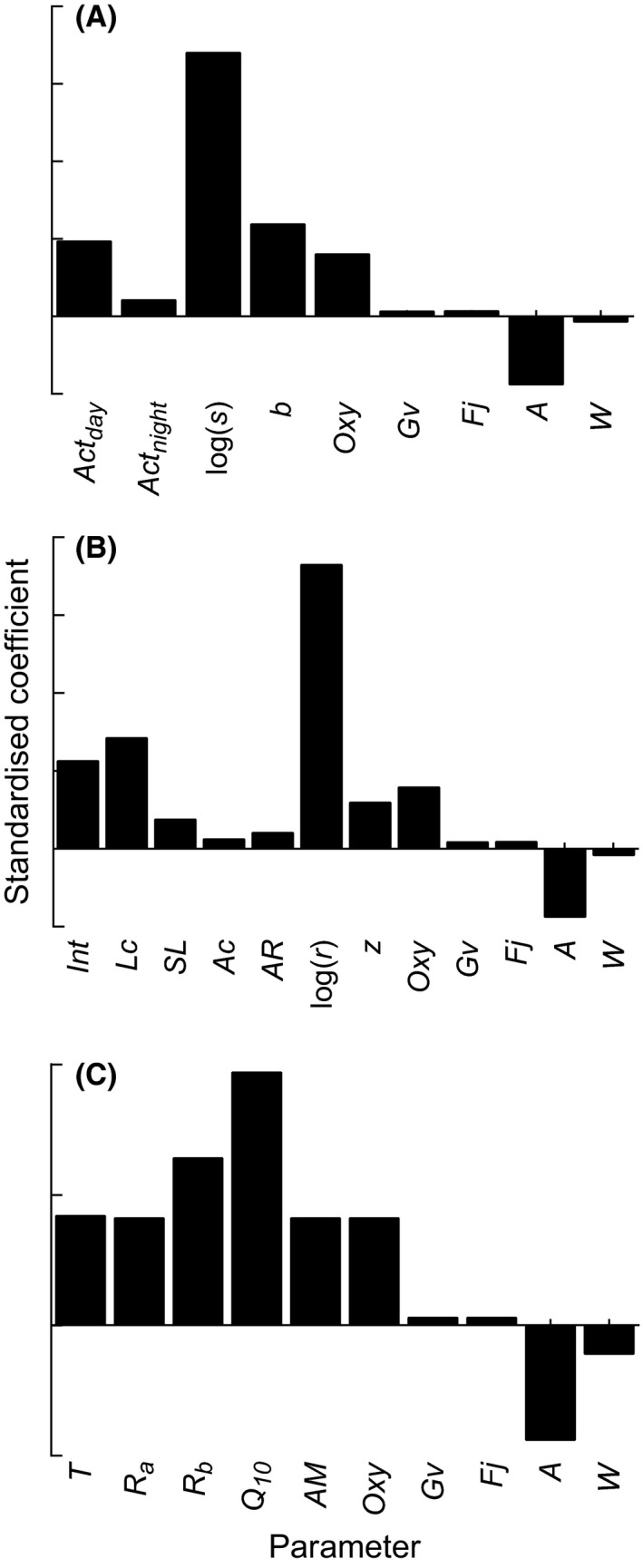
Results of sensitivity analysis for three models that estimate consumption rates of kingfish: (A) accelerometry model; (B) morphometric model; (C) physiological model. Bars are the coefficients of the multiple regression of standardized simulation data, and represent the relative influence of each parameter on modeled consumption rate. See Table 2 for parameter definitions.

Sensitivity analyses identified a single parameter in each of the three models that had a dominant influence on consumption rate (Fig. 3). For the accelerometry and morphometric models, the constants log(*s*) and log(*r*) had the greatest influence on consumption rates, respectively (Fig. 3A, B). These parameters [log(*s*) and log(*r*)] are intercepts of the exponential equations to calculate metabolic rate from activity (*MR_A_*; Figs. 1B, 3A; eq. (1)) and swimming speed (*MR_S_*; Figs. 1A, 3B; eq. (3)). In the physiological model, parameters were more similar in their relative influence on consumption rate, but the *Q*_10_ parameter (the temperature dependence of metabolism) had the greatest influence (Fig. 3C). Weight (*W*) and assimilation (*A*) were the only parameters in each model that were negatively related to consumption rates (Fig. 3). The parameters that had the lowest relative influence on consumption rates were von Bertalanffy growth rate (*Gv*) and energetic density (*Fj*), and this was consistent across all three models.

### Consumption rate simulations

**Figure 4:**
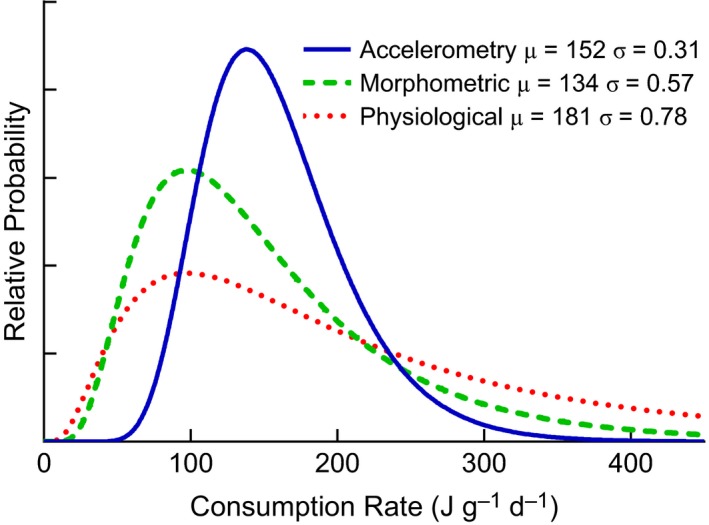
Relative probability of the kingfish consumption (J·g^−1^·day^−1^) estimates from three bioenergetics models, fitted by a log‐normal distribution.

Consumption rate estimates for kingfish were similar between all three models, with the physiological model having the highest estimate (*μ *= 181 J·g^−1^·day^−1^), followed by the accelerometry model (*μ *= 152 J·g^−1^·day^−1^) and then the morphometric model (*μ *= 134 J·g^−1^·day^−1^; Fig. 4). The variance of the log‐normal distribution influences the shape and position of the curves in Figure 4, with smaller variances shifting the peak of log‐normal curve closer to the mean. The accelerometry model estimate had the smallest variance for the log‐normally distributed consumption rate (*σ *= 0.31), compared with the morphometric (*σ *= 0.57) and physiological models (*σ *= 0.78; Fig. 4). The median values of simulated outputs of consumption rates show a similar pattern to mean consumption, with the median accelerometry estimate (*M* = 151 J·g^−1^·day^−1^) greater than the morphometric estimate (*M* = 119 J·g^−1^·day^−1^) and smaller than the physiological estimate (*M* = 178 J·g^−1^·day^−1^).

## Discussion

Incorporating field‐derived activity into a bioenergetics model should provide more realistic estimates of consumption rates compared with traditional methods. The accelerometry method measures real variation in wild body activity, whereas the physiological method assumes fixed activity, and the morphometric method derives activity based on fixed morphological traits. Uncertainty in active metabolic rate can be accounted for in the morphometric method by using the standard error of the empirical regression (Sambilay 1990), but this variation represents intraspecific variation. Neither the physiological nor morphometric methods account for the real between‐ and within‐individual variation in active metabolic rate. The accelerometry method incorporates both these types of variation, which are probably the most relevant to estimating accurate species‐specific consumption rates. Another advantage of the accelerometry method is the ability to partitition active metabolic rate across ecologically relevant scales. In this study, for example, wild kingfish showed strong diel behavior, with mean daytime activity almost double that of mean nighttime activity. The relevant scale at which to partition animal activity can be informed through the behavioral ecology of the study species. For example, fine temporal scales can be used to assess the energetic costs of episodic environmental events (e.g., rainfall; Payne et al. 2013) or diel movement behavior (Gleiss et al. 2013), whereas coarse temporal scales can be used to assess seasonal trends in animal activity (Stehfest et al. 2015). Exploring the variation in active metabolic rate across these scales is not possible in the physiological or morphometric methods.

### Evaluating the metabolic rate equations

The distribution of estimated consumption rates in the accelerometry method had the smallest variance despite this method incorporating the variation of wild activity. This is surprising, given the real variation within and between wild individuals that was incorporated into the accelerometry method, and indicates the level of uncertainty inherent in the other methods. The sensitivity analyses revealed that the parameters associated with estimating metabolic rate had the largest influence on consumption rate estimates. For the accelerometry and morphometric models, the most influential parameters were the intercept of the linear models derived from the laboratory calibrations [log(*s*), log(*r*)]. This occurred despite the morphometric model including a large source of uncertainty from the multispecies empirical regression (eq. (8)). The importance of log(*s*) and log(*r*) parameters in the accelerometer and morphometric models indicates the significance of the functions used to derive active metabolic rate from activity (eq. (1)) and swimming speed (eq. (3)). Log(*s*) and log(*r*) are the random intercepts that included real between‐individual variation in the laboratory calibrations. The between‐individual variation could have been examined by using whole‐animal metabolic rates instead of mass‐specific metabolic rates, which ultimately would have decreased the error of field metabolic rate estimates. However, insufficient field data for the experimental kingfish prevented the use of whole‐animal metabolic rates being used in consumption rate estimates.

The laboratory calibration that derived the relationship between activity (*Act*) and metabolic rate (*MR_A_*) was best described by an exponential equation, as determined from AICc. The exponential form was surprising, given that a majority of studies have found a linear (e.g., Wilson et al. 2013), rather than nonlinear, relationship (e.g., Gleiss et al. 2010; Payne et al. 2011). Gleiss et al. (2010) found that an exponential form of the relationship between body acceleration and metabolic rate could provide better estimates of the standard metabolic rate for hammerhead sharks, as standard metabolic rate was determined by extrapolating the metabolic rate and body acceleration relationship to zero acceleration (i.e., intercept; Gleiss et al. 2010). A linear form of this relationship resulted in a negative standard metabolic rate for hammerhead sharks, with the result attributed to the limited range of laboratory swimming speeds the sharks were exposed to (Gleiss et al. 2010). In this study, the linear form of the kingfish activity and *MR_A_* relationship also had a negative intercept (*MR_A_* = 243 × *Act* − 15), a result that is biologically impossible. As the activity and *MR_A_* relationship (eq. (1)) was being used to predict the field metabolic rate of kingfish, the exponential relationship was kept to improve the predictive power of field estimates and provide realistic metabolic rate estimates at low activity values. The experimental kingfish were swum over a relatively small range of speeds in the laboratory, with field activity values much higher than the laboratory measurements. In order to prevent extrapolation beyond the limits of the laboratory‐derived equation, the mean hourly activity of kingfish was used in consumption rate estimates. The value for both mean hourly activity during the day (*Act_day_*) and night (*Act_night_*) was within range of the activity values recorded in the laboratory calibrations. This ensured that the choice of the exponential form of equation (1) did not provide spurious results, nor inflate the uncertainty of consumption rate estimates.

The sensitivity analysis shows that the temperature dependence of metabolism (*Q*_10_) had the greatest influence on consumption rate estimates in the physiology method. Temperature greatly influences metabolism and activity of animals, yet any scaling relationship between temperature and active metabolic rates remains unclear (Halsey et al. 2015). Integrating field‐derived activity values over a greater temperature range than the range in this study can be done, but the effect of temperature on activity should first be evaluated. This step is critical for highly active animals, such as pelagic fish, where the proportion of energy allocated to active metabolic rate exceeds that of resting metabolic rate (Halsey et al. 2015). Determining the relationship between active metabolic rate and temperature will allow the accelerometry model to be applied to animals exposed to variable environmental temperatures. For active animals, the effect of temperature can be accounted for in the accelerometry model by multiplying the equation for *Temp* (eq. (10)) by the equation for mass‐specific oxygen consumption (*MR_A_*; eq. (1)), where the *Q*_10_ parameter in the *Temp* equation is the rate of change in active metabolic rate as a result of a 10°C temperature increase. For inactive animals, where the proportion of energy allocated to active metabolic rate is small or negligible, environmental temperatures can be incorporated into the accelerometry model by using the *Q*_10_ of resting metabolic rate. This is because, in inactive animals, it can be assumed that the relationship between temperature and active metabolic rate scales similarly to that of temperature and resting metabolic rate (Halsey et al. 2015). For active animals, determining the effect of temperature on active metabolic rate will improve the accuracy of consumption rate estimates, as well as any other behavioral metric that may be affected by temperature.

The high mean consumption rate estimate of the physiological method is partly due to the activity multiplier. Despite the common use of the activity multiplier (Meskendahl et al. 2010; Hughes et al. 2014; Frisk et al. 2015), the assumption that active metabolic rate is a fixed proportion of standard metabolic rate may lead to miscalculations of consumption rates and should be explicitly tested for individual species (Mathot and Dingemanse 2015) and treated conservatively (Meskendahl et al. 2010). The activity multiplier can be estimated from laboratory feeding trials (Madenjian et al. 2012; Cerino et al. 2013); however, the estimated value still remains inflexible in response to changes in environmentally induced fish behavior (Whiterod et al. 2013).

### Application of the bioenergetics models

The three models presented in this study have broad applications, with each model possessing its own advantages and limitations to estimating consumption rates. Accounting for the variation in animal activity levels is one of the key advantages of using the accelerometry method, as fine‐scale temporal variation in consumption rates cannot be resolved using the physiological or morphometric methods. This study used a highly active marine predator to provide an example of incorporating wild activity, but the methods can be applied to species with different behavioral strategies. For example, less active species have a strong accelerometer signal, with transmitters revealing bursts in activity of sedentary species (de Almeida et al. 2013) and ambush predators (Gannon et al. 2014; Landsman et al. 2015). The broad application of the accelerometry method to many aquatic species is a significant advantage; however, the primary limitation of this approach is the requirement of swim‐tunnel respirometry. The use of respirometry to calibrate the metabolic costs of activity can be logistically difficult for large individuals (>10 kg; Payne et al. 2015), or species that require very fast water speeds for minimum swimming requirements (e.g., tuna; Fitzgibbon et al. 2008). For such individuals, the morphometric and physiological methods can provide alternative approaches to estimating consumption rates. The example morphometric approach estimated routine swimming speed by assuming that the caudal fin was the primary mode of locomotion. This approach also required deriving a relationship beween swimming speed and metabolic rate (eq. (3)). In other applications, estimates of optimum swimming speeds based on morphological traits could use theoretical estimates (Weihs 1973; Ware 1978), or other empirical approaches that incorporate alternate swimming modes (Magnuson 1973; Sfakiotakis et al. 1999). The morphometric approach can also use estimates of optimum swimming speed in an empirical model to estimate the energetics costs of swimming (Videler and Nolet 1990; Boisclair and Tang 1993; Ohlberger et al. 2006), which removes the requirement for laboratory calibrations. The advantage of the physiological method is that it can also be used without a corresponding laboratory experiment, but doing so requires the use of existing multi‐species regressions that define the relationship between standard metabolic rate and mass. Incorporating empirical multi‐species relationships into the morphometric and physiological methods allows these methods to be applied to a wide range of species, with no requirement for laboratory respirometry calibrations. However, the use of such empiricial relationships comes with inherent variation, and such uncertainty must be communicated in the final estimates of consumption rates.

A reliable understanding of the trophic demand of predators is needed for ecosystem‐based management. Within the framework of ecosystem‐based fisheries management, innovative methods are required to gain comprehensive information about trophic interactions. For example, the consumption rate estimates reported here have implications for the modeling of energy flow through ecosystems and provide a useful estimate for understanding kingfish production. Kingfish populations are exhibiting a poleward range shift (Robinson et al. 2015) and consumption rate estimates can potentially infer the future predatory impact of kingfish on prey resources, and help promote adaptive management to altered fisheries resources (Koehn et al. 2011). In future, consumption rate estimates for multiple species can be used as a reference point for ecosystem‐based management and could be used to model the implications of altered harvest strategies. Interspecies comparisons revealed similar consumption rates between kingfish (range of method means: 134–181 J·g^−1^·day^−1^) and a comparable piscivorous predator, Arripis *trutta* (102 J·g^−1^·day^−1^; Hughes et al. 2014), but were markedly lower than that of a tuna species that occupies a similar trophic level, *K. pelamis* (465 J·g^−1^·day^−1^; Essington 2003). Future interspecies comparisons could benefit from monitoring differences in fish activity and behavior in the wild. This would help further elucidate the bioenergetics and trophodynamic impacts between fish groups that occupy similar trophic levels. Individual consumption estimates can then be scaled to population consumption by linking bioenergetics models with population models (Essington 2003; Megrey et al. 2007), and using accurate individual‐level data should improve the realism of these population models. There is often high uncertainty in estimates of consumption (as seen in this study); however, incorporating wild activity should ultimately improve accuracy, and the interpretation and application of consumption rate estimates.

## Conflict of Interest

None declared.
